# (*E*)-1-(2,4-Dihy­droxy­phen­yl)-3-(4-hydroxy­phen­yl)prop-2-en-1-one monohydrate

**DOI:** 10.1107/S1600536811006271

**Published:** 2011-02-26

**Authors:** Jian-Guo Wang, Lin Wu, Chan-Juan Zhong, Zhao-Hui Ouyang, De-Lian Yi

**Affiliations:** a Applied Chemistry Research Institute, Wuhan University of Science & Technology, Wuhan 430081, People’s Republic of China; b School of Chemistry & Chemical Engineering, Jiujiang University, Jiujiang 332005, People’s Republic of China

## Abstract

In the title compound, C_15_H_12_O_4_·H_2_O, the two benzene rings are not coplanar, making a dihedral angle of 7.24 (16)°. An intra­molecular hy­droxy–carbonyl O—H⋯O hydrogen bond occurs. In the crystal, four inter­molecular O—H⋯O hydrogen bonds involving the hy­droxy residues, the carbonyl group and the water mol­ecule lead to the formation of a three-dimensional network. The supra­molecular structure is further stabilized by weak C—H⋯O inter­actions.

## Related literature

For the biological activity of the title compound, see: Jang *et al.* (2008 ▶); Liu *et al.* (2008 ▶). For a related structure, see: Ma *et al.* (2005 ▶).
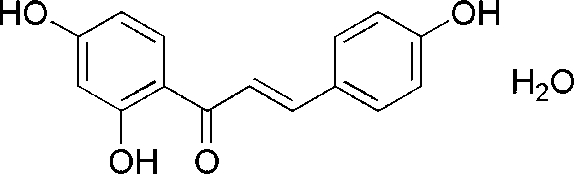


## Experimental

### 

#### Crystal data

C_15_H_12_O_4_·H_2_O
*M* *_r_* = 274.26
Monoclinic, 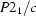

*a* = 11.489 (2) Å
*b* = 9.5903 (17) Å
*c* = 12.498 (2) Å
β = 103.649 (3)°
*V* = 1338.2 (4) Å^3^
*Z* = 4
Mo *K*α radiation
μ = 0.10 mm^−1^
*T* = 298 K
0.12 × 0.10 × 0.10 mm

#### Data collection

Bruker SMART CCD area-detector diffractometer
Absorption correction: multi-scan (*SADABS*; Sheldrick, 1996 ▶) *T*_min_ = 0.988, *T*_max_ = 0.990
8297 measured reflections
2625 independent reflections
2115 reflections with *I* > 2σ(*I*)
*R*_int_ = 0.031

#### Refinement

*R*[*F* ^2^ > 2σ(*F* ^2^)] = 0.057
*wR*(*F* ^2^) = 0.144
*S* = 1.09
2625 reflections
196 parameters
5 restraints
H atoms treated by a mixture of independent and constrained refinement
Δρ_max_ = 0.33 e Å^−3^
Δρ_min_ = −0.19 e Å^−3^

### 

Data collection: *SMART* (Bruker, 1997 ▶); cell refinement: *SAINT* (Bruker, 1997 ▶); data reduction: *SAINT*; program(s) used to solve structure: *SHELXS97* (Sheldrick, 2008 ▶); program(s) used to refine structure: *SHELXL97* (Sheldrick, 2008 ▶); molecular graphics: *SHELXTL* (Sheldrick, 2008 ▶); software used to prepare material for publication: *SHELXTL*.

## Supplementary Material

Crystal structure: contains datablocks I, global. DOI: 10.1107/S1600536811006271/go2004sup1.cif

Structure factors: contains datablocks I. DOI: 10.1107/S1600536811006271/go2004Isup2.hkl

Additional supplementary materials:  crystallographic information; 3D view; checkCIF report

**Fig. 1.**
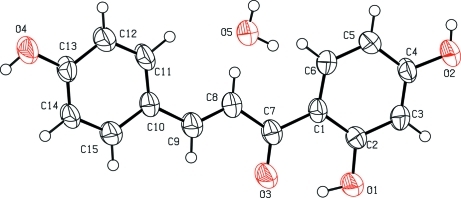
View of the molecule of (I) showing the atom-labelling scheme. Displacement ellipsoids are drawn at the 50% probability level.

Enhanced figure: interactive version of Fig. 1

**Fig. 2.**
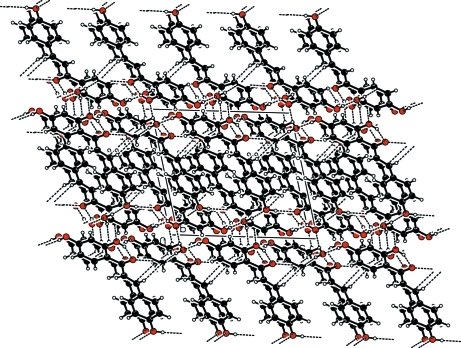
The crystal packing for (I), with O—H···O and C—H···O interactions shown as dashed lines.

Enhanced figure: interactive version of Fig. 2

## Figures and Tables

**Table 1:** Hydrogen-bond geometry (Å, °)

*D*—H⋯*A*	*D*—H	H⋯*A*	*D*⋯*A*	*D*—H⋯*A*
O1—H1*A*⋯O3	0.87 (2)	1.74 (2)	2.530 (2)	150 (3)
O2—H2*A*⋯O5^i^	0.82 (2)	1.83 (2)	2.644 (3)	175 (4)
O4—H4*A*⋯O3^ii^	0.83 (2)	1.95 (2)	2.776 (2)	175 (4)
O5—H5*A*⋯O1^iii^	0.84 (2)	1.99 (2)	2.802 (2)	164 (3)
O5—H5*B*⋯O4^iv^	0.84 (2)	1.97 (2)	2.785 (3)	165 (3)
C9—H9⋯O4^v^	0.93	2.56	3.402 (3)	151

Symmetry codes: (i) 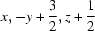; (ii) 
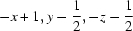
; (iii) 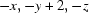; (iv) 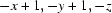; (v) 
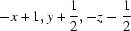
.

## supplementary crystallographic information

### Comment

The title compound exhibits many biological activities such as tracheal relaxation effects (Liu *et al.*, 2008) and suppressing cocaine-induced extracellular dopamine release (Jang *et al.*, 2008).

One (*E*)-1-(2,4-Dihydroxyphenyl)-3-(4-hydroxyphenyl)prop-2-en-1-one molecule bears one crystalline water molecule (Fig.1). In the molecule, the two benzene rings are not coplanar, the dihedral angle being 7.24 (16)°. The structure displays O—H···O and C—H···O hydrogen bonding (Table 1 and Fig. 2).

### Experimental

2, 4-dihydroxyacetophenone (7.6 g, 0.05 mol) and 4-hydroxybenzaldehyde (8.54 g, 0.07 mol) were dissolved in diglycol (25 ml). Then 40% aq. KOH (50 ml) was added, and the reaction mixture was vigorously stirred under nitrogen atmosphere at 333 K for 2 h. The progress of the reaction was monitored by thin- layer chromatography (Si gel, developing solvent *V*(ethyl acetate)/*V*(benzene) = 1:2). The mixture was colled to room temperature and 1:1 (*v*/*v*) hydrochloric acid was added to acidize the mixture to pH=3 and a solid was obtained. After crystallized by ethanol-water, crystalline yellow needles were obtained, m.p. 472.5–474.2 K.

### Refinement

All the carbon-bounded hydrogen atoms were located at their ideal positions with the C—H=0.93Å and *U*_iso_(H)=1.2*U*_eq_(C). All the hydrogen atoms bonded to the oxygen atoms were located from the difference maps and refined with the restraints of O—H=0.82 (1)Å and *U*_iso_(H)=1.5*U*_eq_(O).

### Crystal data

**Table:**

C_15_H_12_O_4_·H_2_O	*F*(000) = 576
*M**_r_* = 274.26	*D*_x_ = 1.361 Mg m^−^^3^
Monoclinic, *P*2_1_/*c*	Mo *K*α radiation, λ = 0.71073 Å
Hall symbol: -P 2ybc	Cell parameters from 2288 reflections
*a* = 11.489 (2) Å	θ = 2.7–25.3°
*b* = 9.5903 (17) Å	µ = 0.10 mm^−^^1^
*c* = 12.498 (2) Å	*T* = 298 K
β = 103.649 (3)°	Block, yellow
*V* = 1338.2 (4) Å^3^	0.12 × 0.10 × 0.10 mm
*Z* = 4	

### Data collection

**Table:**

Bruker SMART CCD area-detector diffractometer	2625 independent reflections
Radiation source: fine-focus sealed tube	2115 reflections with *I* > 2σ(*I*)
graphite	*R*_int_ = 0.031
phi and ω scans	θ_max_ = 26.0°, θ_min_ = 2.7°
Absorption correction: multi-scan (*SADABS*; Sheldrick, 1996)	*h* = −14→14
*T*_min_ = 0.988, *T*_max_ = 0.990	*k* = −11→9
8297 measured reflections	*l* = −15→15

### Refinement

**Table:**

Refinement on *F*^2^	Primary atom site location: structure-invariant direct methods
Least-squares matrix: full	Secondary atom site location: difference Fourier map
*R*[*F*^2^ > 2σ(*F*^2^)] = 0.057	Hydrogen site location: inferred from neighbouring sites
*wR*(*F*^2^) = 0.144	H atoms treated by a mixture of independent and constrained refinement
*S* = 1.09	*w* = 1/[σ^2^(*F*_o_^2^) + (0.0569*P*)^2^ + 0.499*P*] where *P* = (*F*_o_^2^ + 2*F*_c_^2^)/3
2625 reflections	(Δ/σ)_max_ < 0.001
196 parameters	Δρ_max_ = 0.33 e Å^−^^3^
5 restraints	Δρ_min_ = −0.19 e Å^−^^3^

### Special details

**Table:**

Geometry. All e.s.d.'s (except the e.s.d. in the dihedral angle between two l.s. planes) are estimated using the full covariance matrix. The cell e.s.d.'s are taken into account individually in the estimation of e.s.d.'s in distances, angles and torsion angles; correlations between e.s.d.'s in cell parameters are only used when they are defined by crystal symmetry. An approximate (isotropic) treatment of cell e.s.d.'s is used for estimating e.s.d.'s involving l.s. planes.
Refinement. Refinement of *F*^2^ against ALL reflections. The weighted *R*-factor *wR* and goodness of fit *S* are based on *F*^2^, conventional *R*-factors *R* are based on *F*, with *F* set to zero for negative *F*^2^. The threshold expression of *F*^2^ > σ(*F*^2^) is used only for calculating *R*-factors(gt) *etc*. and is not relevant to the choice of reflections for refinement. *R*-factors based on *F*^2^ are statistically about twice as large as those based on *F*, and *R*- factors based on ALL data will be even larger.

### Fractional atomic coordinates and isotropic or equivalent isotropic displacement parameters (Å^2^)

**Table:**

	*x*	*y*	*z*	*U*_iso_*/*U*_eq_	
C1	0.19653 (18)	1.0001 (2)	0.07068 (17)	0.0402 (5)	
C2	0.10947 (18)	1.1026 (2)	0.07420 (17)	0.0412 (5)	
C3	0.0605 (2)	1.1156 (2)	0.16459 (19)	0.0475 (6)	
H3	0.0037	1.1844	0.1655	0.057*	
C4	0.0950 (2)	1.0275 (2)	0.25329 (18)	0.0452 (5)	
C5	0.1818 (2)	0.9256 (3)	0.25306 (19)	0.0500 (6)	
H5	0.2061	0.8663	0.3131	0.060*	
C6	0.2309 (2)	0.9138 (2)	0.16359 (19)	0.0473 (6)	
H6	0.2892	0.8462	0.1644	0.057*	
C7	0.24678 (19)	0.9881 (2)	−0.02538 (18)	0.0437 (5)	
C8	0.3403 (2)	0.8856 (3)	−0.02910 (19)	0.0494 (6)	
H8	0.3588	0.8183	0.0258	0.059*	
C9	0.3990 (2)	0.8858 (3)	−0.10772 (19)	0.0493 (6)	
H9	0.3751	0.9540	−0.1613	0.059*	
C10	0.49523 (19)	0.7959 (2)	−0.12387 (17)	0.0431 (5)	
C11	0.5467 (2)	0.6925 (3)	−0.05077 (19)	0.0566 (7)	
H11	0.5169	0.6760	0.0111	0.068*	
C12	0.6406 (2)	0.6134 (3)	−0.0672 (2)	0.0635 (7)	
H12	0.6750	0.5458	−0.0161	0.076*	
C13	0.68407 (19)	0.6350 (3)	−0.16102 (18)	0.0474 (6)	
C14	0.6341 (2)	0.7353 (3)	−0.23509 (18)	0.0486 (6)	
H14	0.6628	0.7498	−0.2978	0.058*	
C15	0.5412 (2)	0.8148 (3)	−0.21662 (19)	0.0518 (6)	
H15	0.5080	0.8832	−0.2675	0.062*	
O1	0.07099 (15)	1.19360 (19)	−0.01001 (14)	0.0589 (5)	
H1A	0.107 (3)	1.171 (3)	−0.061 (2)	0.088*	
O2	0.04269 (18)	1.0443 (2)	0.33756 (14)	0.0649 (5)	
H2A	0.069 (3)	0.988 (3)	0.387 (2)	0.097*	
O3	0.21067 (14)	1.06876 (19)	−0.10709 (13)	0.0565 (5)	
O4	0.77775 (17)	0.5545 (2)	−0.17385 (14)	0.0668 (6)	
H4A	0.784 (3)	0.563 (4)	−0.2384 (17)	0.100*	
O5	0.12061 (17)	0.6281 (2)	0.00416 (14)	0.0587 (5)	
H5A	0.072 (2)	0.687 (3)	0.017 (3)	0.088*	
H5B	0.149 (3)	0.586 (3)	0.0630 (19)	0.088*	

### Atomic displacement parameters (Å^2^)

**Table:**

	*U*^11^	*U*^22^	*U*^33^	*U*^12^	*U*^13^	*U*^23^
C1	0.0381 (11)	0.0444 (12)	0.0430 (12)	−0.0015 (9)	0.0192 (9)	−0.0060 (10)
C2	0.0413 (11)	0.0446 (12)	0.0415 (11)	−0.0002 (10)	0.0174 (9)	0.0015 (10)
C3	0.0457 (12)	0.0512 (14)	0.0524 (13)	0.0090 (11)	0.0251 (10)	−0.0011 (11)
C4	0.0502 (12)	0.0514 (13)	0.0409 (12)	0.0005 (10)	0.0247 (10)	−0.0039 (10)
C5	0.0583 (14)	0.0530 (14)	0.0436 (12)	0.0078 (11)	0.0217 (11)	0.0070 (10)
C6	0.0485 (12)	0.0485 (13)	0.0497 (13)	0.0086 (10)	0.0213 (10)	−0.0007 (10)
C7	0.0400 (11)	0.0520 (14)	0.0432 (12)	−0.0049 (10)	0.0183 (9)	−0.0041 (10)
C8	0.0513 (13)	0.0551 (14)	0.0483 (13)	0.0061 (11)	0.0249 (10)	−0.0017 (11)
C9	0.0475 (12)	0.0576 (15)	0.0485 (13)	0.0041 (11)	0.0229 (10)	0.0008 (11)
C10	0.0404 (11)	0.0506 (13)	0.0431 (12)	−0.0005 (10)	0.0193 (10)	−0.0044 (10)
C11	0.0605 (15)	0.0757 (18)	0.0437 (13)	0.0113 (13)	0.0323 (12)	0.0049 (12)
C12	0.0690 (16)	0.0791 (19)	0.0495 (14)	0.0290 (15)	0.0285 (12)	0.0156 (13)
C13	0.0425 (12)	0.0596 (15)	0.0446 (12)	0.0065 (11)	0.0193 (10)	−0.0039 (11)
C14	0.0493 (13)	0.0617 (15)	0.0427 (12)	0.0062 (11)	0.0267 (10)	0.0028 (11)
C15	0.0528 (13)	0.0586 (15)	0.0507 (14)	0.0097 (11)	0.0254 (11)	0.0083 (11)
O1	0.0648 (11)	0.0665 (12)	0.0538 (10)	0.0202 (9)	0.0307 (8)	0.0141 (9)
O2	0.0793 (13)	0.0756 (13)	0.0531 (10)	0.0183 (10)	0.0424 (10)	0.0072 (9)
O3	0.0571 (10)	0.0710 (12)	0.0492 (9)	0.0108 (8)	0.0284 (8)	0.0084 (8)
O4	0.0654 (11)	0.0920 (14)	0.0512 (10)	0.0355 (10)	0.0302 (9)	0.0110 (10)
O5	0.0702 (12)	0.0605 (12)	0.0539 (10)	0.0151 (9)	0.0315 (9)	0.0043 (9)

### Geometric parameters (Å, °)

**Table:**

C1—C6	1.405 (3)	C9—H9	0.9300
C1—C2	1.410 (3)	C10—C11	1.383 (3)
C1—C7	1.454 (3)	C10—C15	1.394 (3)
C2—O1	1.359 (3)	C11—C12	1.374 (3)
C2—C3	1.382 (3)	C11—H11	0.9300
C3—C4	1.375 (3)	C12—C13	1.393 (3)
C3—H3	0.9300	C12—H12	0.9300
C4—O2	1.340 (3)	C13—C14	1.364 (3)
C4—C5	1.397 (3)	C13—O4	1.364 (3)
C5—C6	1.371 (3)	C14—C15	1.375 (3)
C5—H5	0.9300	C14—H14	0.9300
C6—H6	0.9300	C15—H15	0.9300
C7—O3	1.270 (3)	O1—H1A	0.867 (18)
C7—C8	1.465 (3)	O2—H2A	0.819 (18)
C8—C9	1.316 (3)	O4—H4A	0.829 (18)
C8—H8	0.9300	O5—H5A	0.836 (18)
C9—C10	1.453 (3)	O5—H5B	0.836 (18)
			
C6—C1—C2	116.61 (18)	C8—C9—H9	115.0
C6—C1—C7	123.2 (2)	C10—C9—H9	115.0
C2—C1—C7	120.24 (19)	C11—C10—C15	117.1 (2)
O1—C2—C3	117.03 (19)	C11—C10—C9	123.66 (19)
O1—C2—C1	121.81 (18)	C15—C10—C9	119.2 (2)
C3—C2—C1	121.2 (2)	C12—C11—C10	121.7 (2)
C4—C3—C2	120.5 (2)	C12—C11—H11	119.1
C4—C3—H3	119.7	C10—C11—H11	119.1
C2—C3—H3	119.7	C11—C12—C13	119.6 (2)
O2—C4—C3	117.5 (2)	C11—C12—H12	120.2
O2—C4—C5	122.6 (2)	C13—C12—H12	120.2
C3—C4—C5	119.90 (19)	C14—C13—O4	122.43 (19)
C6—C5—C4	119.4 (2)	C14—C13—C12	119.9 (2)
C6—C5—H5	120.3	O4—C13—C12	117.6 (2)
C4—C5—H5	120.3	C13—C14—C15	119.7 (2)
C5—C6—C1	122.4 (2)	C13—C14—H14	120.1
C5—C6—H6	118.8	C15—C14—H14	120.1
C1—C6—H6	118.8	C14—C15—C10	121.9 (2)
O3—C7—C1	119.85 (19)	C14—C15—H15	119.0
O3—C7—C8	119.07 (19)	C10—C15—H15	119.0
C1—C7—C8	121.1 (2)	C2—O1—H1A	107 (2)
C9—C8—C7	122.1 (2)	C4—O2—H2A	111 (3)
C9—C8—H8	119.0	C13—O4—H4A	108 (2)
C7—C8—H8	119.0	H5A—O5—H5B	107 (3)
C8—C9—C10	130.0 (2)		
			
C6—C1—C2—O1	178.7 (2)	C2—C1—C7—C8	177.9 (2)
C7—C1—C2—O1	−0.9 (3)	O3—C7—C8—C9	8.8 (4)
C6—C1—C2—C3	−0.5 (3)	C1—C7—C8—C9	−170.2 (2)
C7—C1—C2—C3	179.9 (2)	C7—C8—C9—C10	178.4 (2)
O1—C2—C3—C4	−179.8 (2)	C8—C9—C10—C11	−3.0 (4)
C1—C2—C3—C4	−0.6 (3)	C8—C9—C10—C15	178.3 (3)
C2—C3—C4—O2	−179.0 (2)	C15—C10—C11—C12	1.3 (4)
C2—C3—C4—C5	1.1 (4)	C9—C10—C11—C12	−177.5 (2)
O2—C4—C5—C6	179.5 (2)	C10—C11—C12—C13	−1.5 (4)
C3—C4—C5—C6	−0.6 (4)	C11—C12—C13—C14	0.7 (4)
C4—C5—C6—C1	−0.5 (4)	C11—C12—C13—O4	179.7 (2)
C2—C1—C6—C5	1.1 (3)	O4—C13—C14—C15	−178.9 (2)
C7—C1—C6—C5	−179.4 (2)	C12—C13—C14—C15	0.1 (4)
C6—C1—C7—O3	179.3 (2)	C13—C14—C15—C10	−0.3 (4)
C2—C1—C7—O3	−1.1 (3)	C11—C10—C15—C14	−0.4 (4)
C6—C1—C7—C8	−1.7 (3)	C9—C10—C15—C14	178.4 (2)

### Hydrogen-bond geometry (Å, °)

**Table:**

*D*—H···*A*	*D*—H	H···*A*	*D*···*A*	*D*—H···*A*
O1—H1A···O3	0.87 (2)	1.74 (2)	2.530 (2)	150 (3)
O2—H2A···O5^i^	0.82 (2)	1.83 (2)	2.644 (3)	175 (4)
O4—H4A···O3^ii^	0.83 (2)	1.95 (2)	2.776 (2)	175 (4)
O5—H5A···O1^iii^	0.84 (2)	1.99 (2)	2.802 (2)	164 (3)
O5—H5B···O4^iv^	0.84 (2)	1.97 (2)	2.785 (3)	165 (3)
C9—H9···O4^v^	0.93	2.56	3.402 (3)	151

Symmetry codes: (i) *x*, −*y*+3/2, *z*+1/2; (ii) −*x*+1, *y*−1/2, −*z*−1/2; (iii) −*x*, −*y*+2, −*z*; (iv) −*x*+1, −*y*+1, −*z*; (v) −*x*+1, *y*+1/2, −*z*−1/2.
